# Competition for roadside camera monocular 3D object detection

**DOI:** 10.1093/nsr/nwad121

**Published:** 2023-05-04

**Authors:** Jinrang Jia, Yifeng Shi, Yuli Qu, Rui Wang, Xing Xu, Hai Zhang

**Affiliations:** Baidu Inc., China; Baidu Inc., China; Institute of Artificial Intelligence and Robotics, Xi’an Jiaotong University, China; College of Software, Jilin University, China; School of Computer Science and Engineering, University of Electronic Science and Technology of China, China; Pazhou Laboratory (Huangpu), China; School of Mathematics, Northwest University, China; Pazhou Laboratory (Huangpu), China

## INTRODUCTION

Accurate environment perception is a critical topic in autonomous driving and intelligent traffic. Current environmental perception methods mostly rely on on-board cameras. However, limited by the installation height, there are problems such as blind spots and unstable long-range perception in vehicle perceptual systems. Recently, with the rapid improvement of intelligent infrastructure, it has become possible to use roadside cameras for traffic environment perception. Benefiting from the increased height when compared with on-board sensors, roadside cameras can obtain a larger perceptual field of view and realize long-range observation.

Roadside perception is of great significance to autonomous driving and traffic intelligence. With the help of roadside perception, autonomous vehicles can achieve a global perspective far beyond the current horizon and covering blind spots that can greatly increase safety. Since information from roadside cameras can broadcast to all surrounding autonomous vehicles, the additional economic cost brought about by roadside equipment is worthy. What is more, roadside perception can also achieve traffic flow control and improve efficiency. In order to achieve roadside perception, high-precision roadside monocular three-dimensional (3D) detection algorithms are essential.

### Challenge

Roadside monocular 3D detection has the following challenges.

- *Domain gap.* Compared with the relatively fixed installation parameters of on-board cameras, each roadside camera has various specifications, such as camera intrinsic, pitch angle and mounting height, which introduces domain gaps. Tasks that involve domain gaps pose additional challenges to roadside 3D detection, due to the inherent ambiguities.
- *On-board prior invalid.* The roadside camera is installed on a pole with a pitched angle, so the on-board prior assumption that the optical axis of the camera is parallel to the ground is no longer valid, and thus monocular 3D detection methods with this prior cannot be directly applied.
- *More obstacles.* The utilization of roadside cameras enables the perception system to apprehend a wider range of obstacles, resulting in an increase in obstacle density. As a result, the challenge faced by the perception system becomes increasingly complex.
- *Generalization.* Generalization of the roadside algorithms is difficult. Compared with autonomous driving vehicles that move everywhere, roadside cameras are fixed in position after installation and can only collect data from a single scene. The long-tail problem faced by roadside perception is more serious.

In order to solve the above problems and promote roadside perception to assist intelligent transportation, this competition focuses on roadside monocular 3D detection tasks. Based on roadside image data and labeling information, participating teams designed monocular 3D detection algorithms to achieve the predictions of object category, 2D bounding box, location, dimension (length, width and height) and orientation in roadside area of interest.

### Competition details

#### Dataset

**Figure 1.**
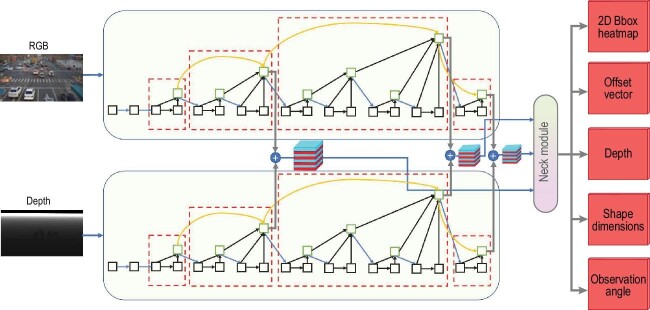
The dual-stream model structure consists of separate feature extraction modules for the depth and RGB images.

The competition used the Rope3D [1] benchmark to evaluate algorithms, which consists of camera images, intrinsics, extrinsics, ground norm files, depth maps and annotations. The dataset provided a total of 50 000 training images with 1920 × 1080 resolution. As shown in Fig. 1 within the online supplementary material, it contained scenes with different weather conditions (sunny, cloudy, rainy), different times (daytime, night, dawn/dusk) and different densities (crowded, normal, less traffic). There were a total of 13 classes annotated: car, van, truck, bus, pedestrian, cyclist, motorcyclist, barrow, tricyclist, traffic cones, triangle plate, unknown unmovable and unknown movable. The distance between obstacles and corresponding cameras ranged from 10 to 140 m. More than half of the objects were partially or heavily occluded.

#### Evaluation metric

Following Rope3D, the competition used *Rope_score_* as the main metric that consists of five submetrics, such as average precision (AP), average ground center similarity (ACS), average orientation similarity (AOS), average area similarity (AAS) and average four-ground-point distance and similarity (AGS). The form of *Rope_score_* is


(1) \begin{eqnarray*} Rope_{score}=\frac{\omega _1 * AP + \omega _2 * S}{\omega _1 + \omega _2}, \end{eqnarray*}

where *S* = (*ACS* + *AOS* + *AAS* + *AGS*)/4, ω_1_ = 8, ω_2_ = 2. More details of the submetrics can be found in ref. [1].

## THE SOLUTION OF THE WINNING TEAM

### Baseline selection

This competition based on the Rope3D dataset is essentially a monocular 3D detection task. Many mature and excellent on-board methods of this field have been proposed in recent years [2,3]. However, most of these approaches are based on the assumption that the optical axis of the camera is parallel to the ground. Roadside cameras do not satisfy such an assumption, leading to an inability to apply these methods directly. Additionally, some approaches [4,5] relied on LIDAR data to provide additional modalite information to assist learning, and thus cannot be used in this competition. Among the remaining methods, MonoDLE [6], which is based on CenterNet [7], considers both performance and runtime. It proposes a 3D intersection-over-union-based dimension loss and uses the *Laplace* method to model depth uncertainty to further improve the accuracy of 3D attribute prediction. As a kind of 3D detection paradigm, MonoDLE has high scalability and does not rely on any strong prior assumptions. Based on the above analysis, MonoDLE was chosen as the baseline.

### Method design

Compared to on-board detection, one of the difficulties of roadside problem is the diversity of camera configurations. For instance, the focal length ranges from 2200 to 3000 pixels in the Rope3D dataset, which may cause obstacles with different depths under different focal lengths corresponding to similar appearance features. This makes it ambiguous to estimate the depth of an obstacle directly from a single RGB image. In order to address this problem, a depth map was introduced as auxiliary data. Furthermore, a variety of depth information utilization strategies were developed, and finally, a dual-stream network was designed. Experiments proved its effectiveness for roadside monocular 3D detection. Different hyper-parameter settings were also fully explored to improve the model performance. Overall, the contributions are summarized as follows.

- In order to solve the domain gap problem of roadside images, depth maps were introduced to eliminate ambiguity.
- Different strategies of utilizing depth maps were explored and a final dual-stream network structure was designed.
- Under comprehensive parameter tuning, the final model achieved smaller floating-point operations per second (FLOPs) while maintaining outstanding precision performance.

### Details

Using a depth map can make the monocular 3D detection task become unambiguous under different focal lengths. Essentially, after the introduction of depth information, the monocular 3D detection task was transformed from an ill-posed problem to a complete optimization problem. The model can use depth information to fine-tune *z* to achieve more accurate 3D detection.

However, how to make better use of depth information is key to further improving performance. One of the most typical and straightforward ideas is concating the depth map and RGB image along the channel dimension and feeding them to the model to extract features. Another method is also based on shared weights, but the depth map and RGB image can be fed into the network separately, and then their features can be fused. A better technique is to build a separate feature extraction module for the depth image, and then fuse the depth features and RGB features in different stages. Our experiments demonstrated that this technique can achieve better performance.

The model can be trained end-to-end and the whole pipeline is as follows: (1) feature extraction, (2) feature interaction and fusion, (3) regression head, (4) loss calculation and backpropagation. The DLA34 [8] network was chosen as the feature extraction backbone, as it provides the most balanced trade-off between speed and precision. Depth maps and RGB images have two separate feature extraction modules. At each stage of the backbone, feature maps of the depth and RGB images were fused. Considering the FLOPs of the model, an adding operator was used. The simplified model structure is shown in Fig. 1.

### Experiments

This subsection presents the details of the experiments. For fair comparison, no pretrained models were used. Table 1 in the online supplementary material shows the key results of the model in the competition.

- *Hyper-parameter choice.* The AdamW optimizer performed best in the model and the batch size was set at 16. Meanwhile, the warm-up technique and cosine warm-up were used in the training stage where the initial learning rate was 2 × 10^−3^.
- *Data processing.* Instead of forcing the model to focus on the hard samples, the proposed method ignored some extremely small and far cases, leading to an improvement in the overall performance. Image augmentation techniques such as horizontal mirror flip, brightness contrast, Gaussian noise and motion blur were also used to improve the diversity of data.
- *Image scale.* Generally, the test performance is sensitive to scale changes of the input image. A larger scale can usually achieve better performance, but it is subject to the law of diminishing marginal utility. The main reason lies in the fact that score-scale variation patterns between different categories (car, big vehicle, cyclist and pedestrian) are different. Thus, finding an appropriate scale was crucial, taking into account FLOPs and training resource balance. In this solution, an input size of 768 × 480 performed better than 960 × 544. The former could train the model for 20 epochs, while the latter could only train for 10 epochs. In the absence of pretrain, the number of iterations can directly affect the convergence of the model.

Parts of some important ablation experiments are listed in Table 2 of the online supplementary material. As can be seen from the experimental results, the efficient utilization of depth information is crucial. Concurrently, the balance between the scale and maximum epoch is also a key improvement point. Consequently, hyper-parameter tuning helped the model achieve better performance. Additionally, the competition used a 2D region of interest (ROI) to filter out obstacles. The score of the model in the ROI was $57.68\%$, which was the highest score.

## COMMENTS ON THE CHAMPION’S SOLUTION

The champion method achieves the highest *Rope_score_* in this competition. Firstly, it introduces a depth map to tackle the domain gap problem in roadside 3D detection. Meanwhile, since monocular depth estimation is actually a more ill-conditioned problem, the depth map can also provide a strong depth prior, making obstacle depth estimation easier. Furthermore, it explores different strategies that focus on how to use depth data. Based on theoretical analysis and a large number of experimental results, the method uses two independent feature extraction backbones and performs feature fusion in multiple stages. Compared to concat depth maps and RGB images directly, this structure has advantages in feature extraction of different modalities. Additionally, horizontal mirror flip and some photometric data augmentation methods are utilized to enrich samples. Compared with some data enhancement methods that may introduce feature ambiguity (such as copy-paste, random crop-expand, camera movement, etc.), horizontal flip and photometric data enhancement can improve the effect without side effects.

Overall, the champion method achieved state-of-the-art performance while maintaining a fast speed. As an end-to-end approach, it is convenient for application in industry.

## CONCLUSION AND FUTURE DIRECTION

The competition aims to promote the development of roadside 3D monocular detection algorithms. Many teams have designed different solutions according to the challenges of roadside perception. Although these methods have achieved excellent performance, there are still improvements for future research. Some possible optimization directions and suggestions are as follows.

- *Cross domain.* In industrial applications, the wide deployment of roadside cameras will introduce a more serious domain gap due to various camera focal lengths and divergences in installation settings, which increases the difficulty of depth estimation. Encoding this information into the model may be helpful to alleviate this problem explicitly. Conversely, guiding models to learn related features to implicitly adapt to different domains is also a line of thought.
- *Geometric modeling.* The champion method used the depth map as additional information. Each depth map is fitted from a single ground plane. However, due to the fact that the ground may have slopes and undulations, it is difficult for a single ground plane to precisely describe the physical characteristics that may cause a decrease in accuracy. Thus, how to model the ground more accurately is also a topic deserving of inquiry. In addition, analogous to the near-large and far-small imaging model of vehicle-side perception, the roadside algorithm can also summarize the prior knowledge, and establish a corresponding mathematical model to further optimize perception.
- *Global information.* Because of the static nature of roadside cameras, it is possible to design a method that can conveniently and effectively utilize global features to enhance the ability of depth estimation.
- *Data augmentation.* Roadside perception is limited by the difficulty in collecting diverse data, and the long-tail problem is harder to solve than the on-board problem. Data augmentation is considered one of the most effective ways to improve generalization. However, besides photometric distortion and random horizontal flipping, most 2D data augmentation methods introduce ambiguity to 3D detection due to breaking the imaging principle. Thus, designing 3D data enhancements without introducing ambiguous features for roadside perception is an urgent problem to be solved.

## Supplementary Material

nwad121_Supplemental_File
