# Supplementary material for: Competition for roadside camera monocular 3D object detection
Source: Natl Sci Rev. 2023 May 4;10(6):nwad121. doi: 10.1093/nsr/nwad121 (PMC10246814; doi:10.1093/nsr/nwad121)
Supplement: nwad121_Supplemental_File [file nwad121_supplemental_file.pdf]

APPENDIX

1 Results of the winning team.

Table 1 shows the main results of the winning team, and Table 2 shows the benefits of different strategies under different parameter configurations. From the first and third rows of Table 2, it can be seen that after introducing depth information, the score has increased by 5.42%. After using hyper-parameter tuning, the score further improved by 1.14%. Finally, in the case of using 2D ROI (as shown in Figure 3), the score achieved 57.68%.

Table 1: Our achievements

Round	Public	Private	Rank
Preliminary	0.4526	0.4586	1
Final	0.5768	0.5302	1

Table 2: Some valuable experimental results

Experiment settings	ROI	Public Score
baseline(768x480 + 20e)		0.4392
960x544 + 10e + depth		0.4846
768x480 + 20e + depth		0.4934
768x480 + 20e + depth + hyper-parameter tuning		0.5048
768x480 + 20e + depth + hyper-parameter tuning	✓	0.5768(final score)

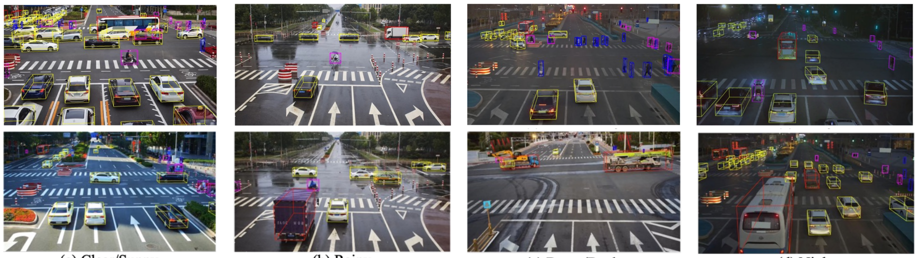

(a) Clear/Sunny (b) Rainy (c) Dawn/Dusk (d) Night

Figure 1: Examples of training data used in this competition under different conditions. From left to right, each column corresponds to clear/sunny, rainy, dawn/dusk and night.

2 Details of the data used in the competition.

The different conditions. As shown in Figure1, the data used in the competition contained scenes with different weather conditions (sunny, cloudy, rainy), different times (daytime, night, dawn/dusk) and different densities (crowded, normal, less traffic).

The ambiguity analysis. Figure 3 shows that the roadside dataset is inherently ambiguous due to the use of cameras with varying focal lengths (2200 to 3000 pixels), mounting heights (6.4m to 7.6m), and pitch angles (9 degrees to 14 degrees), which increases the difficulty of the task.

Depth distribution. The dataset contains 3D objects with depths ranging from 10m to over 140m, with the majority falling between 60m and 80m.

Occlusion and truncation Analysis. Among the data used in the competition, more than 50% of the obstacles are partially or heavily occluded, which increases the difficulty of the competition.

ROI. Obstacles in the white region are used to compute score.

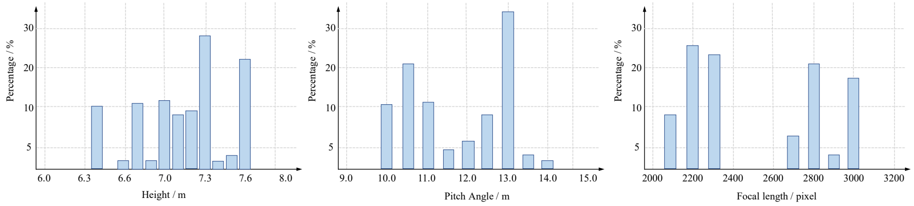

(a) (b) (c)

Figure 2: The diversity of roadside cameras. From (a) to (c) are the distribution of mounting heights, pitch angles of the cameras and the focal lengths over the dataset, respectively.

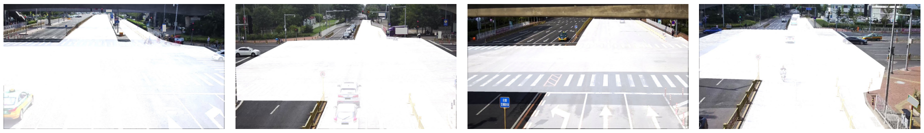

(a) (b) (c) (d)

Figure 3: 2D ROI used in the competition.
